# Prediction Algorithm of User's Brand Conversion Intention Based on Fuzzy Emotion Calculation

**DOI:** 10.3389/fpsyg.2022.907035

**Published:** 2022-05-26

**Authors:** Youwen Ma

**Affiliations:** School of Media and Communication, Shanghai Jiao Tong University, Shanghai, China

**Keywords:** prediction algorithm, brand conversion, fuzzy emotion calculation, brand intention, branding

## Abstract

Branding is a magic weapon for enterprises to participate in international competition, and empowering enterprises through branding has become a national strategy in the new era. Economic and social development has won wide acclaim from the international community, but enterprises generally have the problem of being “big but not strong”, which is not matching with long history and great power influence. The brand bottleneck of Chinese enterprises has been highlighted. Recent brand theory research has been fruitful on the whole, but there are also some weak links, among which “the mechanism of enterprise brand value formation” is a research theme to be strengthened. This paper presents a number of suggestions for the formation of corporate brand value. The empirical analysis was conducted using valid data. The results found that: customer involvement behavior has a significant positive influence on customer citizenship behavior and customer experience value. Customer experience value has a significant positive influence on customer satisfaction and customer commitment. It plays a mediating effect in the relationship between the influence of customer involvement behavior on customer satisfaction and customer commitment, respectively. Customer satisfaction has a significant positive influence on customer commitment, and plays a mediating customer commitment has a significant positive effect on customer citizenship behavior and mediates the effect of customer experience value on customer citizenship behavior. The experimental results show that: the accuracy of crop color recognition by this method is high, and it has the advantages of faster computational efficiency and higher computational accuracy compared with other algorithms, thus verifying the reliability of the algorithm. Based on the fuzzy sentiment of online reviews, this paper improves the continuous use model ECM-ISC and formulates the inference rules of fuzzy affiliation function, and verifies the brand conversion intention and brand conversion type of cell phones by example calculation, which has good accuracy and generality and has important practical significance for brand marketing and early warning management. In addition, the use of brand economics in the study of corporate brand positioning is a development and innovation of brand economics.

## Introduction

In today's international market competition, brand has been regarded as a common language for enterprises to participate in international market activities, and is an important manifestation of the competitiveness and influence of enterprises in the international market (Wardani and Anwar, 2019). With the development of the world economy and the enhancement of enterprises' competitive consciousness, international market competition has moved from primary commodity competition to higher brand competition, from pursuing traditional price advantage and quality advantage to emphasizing brand advantage (Wu et al., 2020). The applicability and practicality of the international market competition model represented by “manufacturing, branding, and low price” is getting less and less, and the disadvantages of this model are becoming more and more obvious, and the sustainability is getting weaker and weaker (Yeh et al., 2018). While the enterprises that rely on brand advantages to participate extensively in international market competition are taking advantage of it, showing strong vitality and growth potential. More and more companies are focusing on the important role of customers, and they want them to be more proactive in their activities, to cooperate with them, and to create value together (Bae et al., 2019). Virtual brand communities provide such a platform (Campbell and Singh, 2019; Indiani and Fahik, 2020; Dangi et al., 2021). By involving customers in the topics and creative calls that companies organize and initiate in the communities, companies can not only get a detailed and comprehensive understanding of their customers' needs for products or services, but also improve the efficiency of production innovation and product quality (Kumar and Nayak, 2018).

These are influenced by the process of customer participation in activities in the community, and also by the customer's own experience, psychology and behavior during this participation process, such as customer satisfaction, customer commitment, etc. (Liu and Ling, 2019). By exploring the transformation mechanism between different types of customer value co-creation behaviors in virtual brand communities, we clarify the influencing factors between behaviors, as well as the changes in cognition, emotion and attitude of customers in the process of manifesting one behavior to another, which is conducive to the timely adjustment of relationship marketing strategies and the identification of problems in customer management, so that companies can take corresponding measures to guide, intervene and influence customers to behave in a way that is beneficial to the company (Madbouly et al., 2020; Prajogo and Purwanto, 2020; Nixon and Steuber, 2021). The active cooperation between customers and enterprises in the process of value co-creation based on virtual brand communities can, on the one hand, enable customers to obtain multiple experience values and, on the other hand, enable enterprises to maintain a high level of relationship quality with customers while satisfying their needs and gaining a sustainable competitive advantage (Putri, 2021). Theoretical research should be about the establishment of a model of corporate brand value formation in the formation mechanism of corporate brand value. At the same time, theoretical research on corporate branding has never stopped, and the theoretical research on corporate branding has been continuously enriched and deepened. On the one hand, the theoretical innovation of corporate branding is carried out to guide corporate branding practice, and on the other hand, the practice of corporate branding is sublimated to enrich corporate branding theory (Rahmi et al., 2022). The theoretical research on this sub-topic needs to be strengthened (Rup et al., 2021). In this paper, we will summarize the current situation of theoretical research on “corporate brand value formation” in detail in the literature review section. This paper discusses the global background, national background and current background of the formation mechanism of corporate brand value. Clearly defines the connotation and composition of corporate brand and corporate brand value (Wu et al., 2019; Shanmuga Sundari and Subaji, 2020; Saleem et al., 2021). Systematically and deeply studies the formation mechanism of corporate brand value, explores the factors and processes influencing the formation of corporate brand value, conducts theoretical research on the formation mechanism of corporate brand value, establishes the model of corporate brand value formation and conducts empirical research on the formation mechanism of corporate brand value (Zhou et al., 2019).

The research will be combined with the analysis system of the factors and processes of corporate brand value formation, and the BPCI theoretical model of corporate brand value formation will be established. The main research contents include: research on the mechanism of creating corporate brand value based on corporate brand. Research on the mechanism of deriving corporate brand value based on corporate brand positioning. Research on the mechanism of adding value to corporate brand value based on corporate brand communication. Research on the mechanism of regulating corporate brand value based on corporate brand innovation. This paper analyzes the formation of corporate brand value by using the root study method, finds the main factors influencing the formation of corporate brand value, constructs a theoretical model of corporate brand value formation and conducts preliminary theoretical discussions. This paper verifies the mechanism of corporate brand value formation through empirical research, case studies, theoretical studies and literature studies.

It constructs a BPCI theoretical model of corporate brand value formation, a corporate brand positioning model based on brand economics, a Bass model of corporate brand communication, a Bass model based on corporate brand communication, and a Bass model based on corporate brand communication. The BPCI theoretical model of corporate brand value formation, corporate brand positioning model based on brand economics, corporate brand positioning model, corporate brand communication Bass model, and quantitative model with corporate brand innovation as the regulating variable are model innovations in the study of corporate brand value formation.

## Methods

### User Brand Switching Intention Model Construction

**Figure 1:**
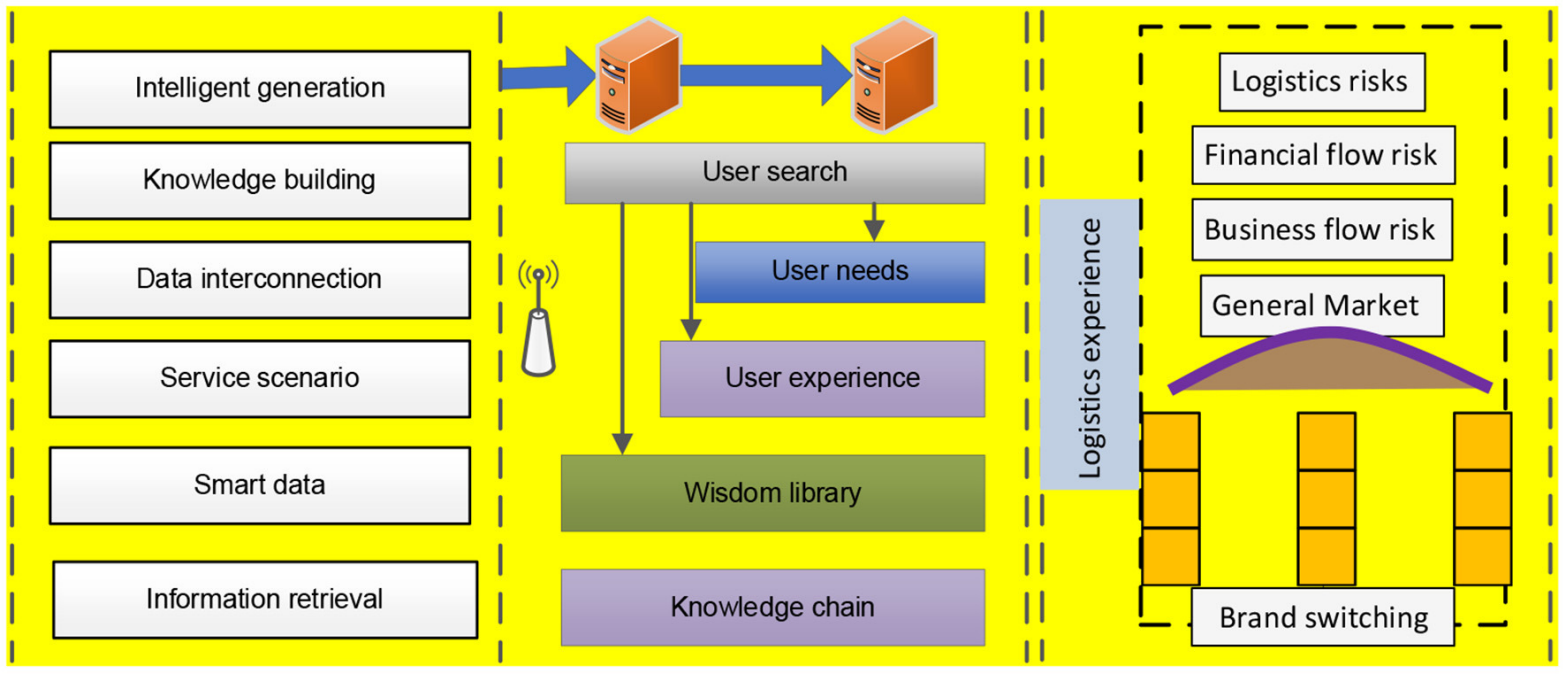
Brand switching intention model.

Consumers' experience with online products and service perceptions are the antecedents of brand switching, and the consumer satisfaction index is usually used to measure users' intention to switch brands. It is known that consumers' brand switching is the change of continuous use intention; therefore, consumers' brand switching intention can be measured by users' continuous use intention. For the measurement of persistent use intention, the information system persistent use model (ECM-ISC), modified from ECM-ISC, is shown in Figure 1.

Satisfaction is the basis of continued use of the brand, which is the overall emotional evaluation after comprehensive use. In this paper, we use the improved continuance model ECM-ISC as the basic theoretical reference to explore the switching intention of consumers' online reviews. Perceived quality, perceived value and perceived service are the most important antecedents of continued use. The contributing factors are the external factors of consumers' intention to continue using the brand, including product promotion, product renewal, etc..

In the process of using the model research method, the following two points are crucial to the accuracy and scientific validity of the research:

1. Construction of fuzzy sentiment lexicon for online product reviews. The fuzzy sentiment dictionary can effectively categorize the review attributes and sentiment polarity.
2. Fuzzy calculation of users' brand switching intention. Online product information and service attributes are reflected in consumers' online reviews, and the scientific measurement algorithm to fully measure and evaluate the valid information of all reviews is the basis of scientific and continuous research.

### Fuzzy Clustering Algorithm

When making intelligent judgments and responses to the environment, image clustering and fuzzy algorithms based on emotion computing can be used to improve their efficiency in processing complex environmental data. Currently, most of the intelligent computing models use distributed computing methods, and the data sources of distributed computing are not uniform. The emotion modules obtained using computer systems need to be expressed with the help of machine language, and programming techniques are used to efficiently execute the machine language, which is the focus of the design of intelligent recognition models for emotion computing. AML (Avatar Markup Language) is an XML-based multiform scripting language, which has a simple structure, is easy to understand, and has good compatibility with various software, and can be used to successfully implement the expression of emotions in the environmental factors of picking operations. To realize the intelligence of the picking robot it is not enough just to use a simple machine language, but also with the help of intelligent algorithms. A good model of intelligent algorithm is the fuzzy c-mean clustering algorithm (FCM), which is a relatively simple procedure. This algorithm can classify data intelligently based on the affiliation of the collected data, and is an improvement of the hard c-mean clustering (HCM) method, first proposed by Bezdek. FCM algorithm uses the fuzzy grouping method, divides n vectors X_i_ (i = 1, 2, ..., n) into c groups, and finds the cluster center of each group, and the affiliation matrix u is allowed to have elements that take values between 0, 1. By normalization, it is specified that the sum of the affiliation degrees of a data set is always equal to 1. The expression is:


(1) $$\begin{array}{l} \overset{n}{\underset{i=1}{\sum }}u_{ij}=1,i,j=1,...,n \end{array}$$

Then the objective function of FCM can be obtained as:


(2) $$\begin{array}{l} J\left(U,c_{1},c_{2},..,c_{n}\right)=\overset{n}{\underset{i=1}{\sum }}{u_{i}}_{j} \end{array}$$

where c_i_ denotes the cluster center of fuzzy group I; d_ij_ = ||c_i_-x_j_|| is the Euclidean distance between the Ith cluster center and the Jth data point, and m ϵ [1, ∞) is a weighted index. The necessary condition to minimize equation can be found by constructing a new objective function as follows:


(3) $$\begin{array}{l} J\left(U,c_{1},c_{2},..,c_{n},\lambda_{1},\lambda_{2},..,\lambda_{n}\right)=\overset{n}{\underset{i=1}{\sum }}{u_{i}}_{j}{d_{ij}}^{2}+\lambda \left(\overset{n}{\underset{i=1}{\sum }}{u_{i}}_{j}-1\right) \end{array}$$

### Product Reviews Online Fuzzy Emotion Dictionary Construction

The fuzzy sentiment lexicon includes basic sentiment lexicon and extended sentiment lexicon. The extended lexicon includes dynamic sentiment lexicon, value evaluation lexicon and sentiment modification lexicon, in addition, based on the semantic complexity of online reviews, a total of about 1,600 extended sentiment words were selected by combining online sentiment words and shopping sentiment words. The construction method and algorithm of the extended sentiment words are as follows:

#### Dynamic Emotion Dictionary

Dynamic emotion words are dynamic words that have the tendency to change the emotion of a phrase, i.e., emotion words that change their emotional attitude when the target of the emotion word being modified changes. The target word modified by a dynamic emotion word determines its emotional tendency. For example, the emotion word “fast”, in the comments of “fast logistics” and “fast operation”, expresses a positive attitude of approval and appreciation, but in the same target comment word However, in the same target comment “fast power consumption”, it has a pejorative tendency. The dynamic sentiment dictionary standardizes a new dynamic polarity calculation criterion, which considers the evaluation target and dynamic sentiment words as a whole, in order to obtain a higher accuracy rate. First, the set of dynamic emotion words is represented by DE. According to the positive and negative attributes of affective tendency, the positive attribute words are represented by positive dynamic set PDE, which consists of dynamic words such as {high, fast, more......}. the negative attribute words are represented by negative dynamic set NDE, which consists of dynamic words such as {low, slow, less......} The negative dynamic set is represented by the negative dynamic set NDE, which consists of dynamic words such as {low, slow, less}. They satisfy the following relationship:


(4) $$\begin{array}{l} PDE⋂NDE=\Phi \end{array}$$

Secondly, for the sentiment tendency and polarity values of the subject word t are denoted by P_t_ and I_t_, respectively, and the dynamic sentiment tendency values of t (P_t_, I_t_) are computed using the pointwise mutual information-based sentiment tendency algorithm SO-PMI, and the rules for computing the dynamic sentiment word and sentiment polarity pairings are as follows:


(5) $$\begin{array}{l} DP(w,t)=\{\begin{array}{l} P=P_{t},w\notin PDE\\ P=-P_{t},w\in PDE \end{array} \end{array}$$

#### Value Evaluation Dictionary

The value evaluation word is the actual evaluation perception sentiment word of consumers' brand purchase, including four attributes: expectation evaluation, perception evaluation, contributing factor evaluation and satisfaction evaluation. The value evaluation sentiment lexicon is based on the combination of online sentiment words and shopping sentiment words, and the value evaluation word polarity sub-criteria with reference to the literature, combined with the ECM-ISC model influencing factors, after manual screening and filtering, the fuzzy value evaluation lexicon example is shown in Table 1.

**Table 1:** Example of value evaluation dictionary statistics.

**Expectation level**	**Expectation evaluation**	**Evaluation of perception**	**Satisfaction**
4	Perfect	First choice	Recommend
3	Love	Trust	Satisfaction
2	Like	Recommend	Pleasant
1	Conformity	Support	Not bad
0	General	General	still acceptable
1	No feeling	Poorly	Dissatisfaction
2	Difference	Regret	Disappointed
3	Error	Collapse	Resist

#### Emotional Modification Dictionary

Emotional modifiers are a collection of adverbs that modify emotionally oriented words, including negative and degree adverbs. Affective modifiers play a key role in commenting on the emotional polarity of a text. Take the emotion word “satisfied”, “dissatisfied” and “especially satisfied” as example, if we ignore the characteristics of the emotion modifier and the context of the comment and consider only the emotion tendency of “satisfied” and “satisfied”. “Satisfaction” without considering the related adverb, it will lead to the error of affective tendency and distortion of affective polarity. In this paper, we combine the semantic features of online reviews, and after machine filtering and manual filtering, we obtain the sentiment modifier lexicon as shown in Table 1.

### Fuzzy Calculation of Users' Brand Switching Intention

Therefore, in order to eliminate the influence of sales volume, this paper conducts cluster analysis by brand conversion intention and monthly sales volume of products, sets the threshold value of monthly sales volume and conversion intention according to the results of cluster analysis, divides the brand conversion type into 4 categories, and further refines the brand conversion type from two dimensions of monthly sales volume and brand conversion intention.

First, the online comment utterances were factorized and syntactically analyzed using the Language Technology Online Presentation Platform (LTP) of the Information Retrieval Research Laboratory, and the evaluation words of all four index factors in the ECM-ISC model were filtered out and classified, and each dictionary was ranked according to the index attributes of the value evaluation factors according to the value evaluation dictionary construction method and ranking rules in Section Validity Analysis, and the corresponding degree adverbs, negative adverbs and other modifiers. The TF-IDF (Term Frequency-Inverse Document Frequency) algorithm is a commonly used method to calculate the weight of feature items, which reflects the importance of a feature factor to a document in a document collection. The calculation method is shown below:


(6) $$\begin{array}{l} W_{i,j}=TF_{ij}\times \frac{\log\left(N/D\right)}{F_{i}} \end{array}$$

Where, W_ij_ denotes the weight of the i_th_ feature factor in text j, TF_ij_ denotes the frequency of the ith feature factor evaluation word in text j, N denotes the total number of online reviews, and DF_i_ denotes the number of texts containing the feature word of the i_th_ factor.

The fuzzy sentiment dictionary corresponds to the fuzzy affiliation function level by intensity, let w be the fuzzy set, and improve the Gaussian function fuzzy calculation method, the fuzzy affiliation function of the 8 sentiment levels of the fuzzy evaluation set is expressed as follows:


(7) $$\begin{array}{l} T_{x}=\frac{\exp\left(\frac{{\left(x-\mu_{w}\right)}^{2}}{2\rho }\right)}{\sigma \sqrt{2\pi }} \end{array}$$

The Fuzzy Inference System (FIS) sets the computational rules, generates all the results from the superposition of inference processes, defuzzifies them by the center-of-mass method, and outputs the posterior values. In this paper, we use expectation evaluation, perception evaluation, contributing factors and satisfaction variables as inference antecedents and brand switching intention as inference postecedents, and the four antecedents are in the range of [−1,4] and the postecedents are in the range of [−6,16], and a total of 584 rules are established in the calculation process. According to the behavior of online consumers, the higher the value of online users, the stronger the recognition of the product, and the lower the intention of brand switching, so the fuzzy inference rules are set as the latter part of the model decreases with the increase of the former part.

The influence factor index of each attribute is set as Ri, and the influence factor index weight, satisfying w ≥ 0, is calculated as follows:


(8) $$\begin{array}{l} R_{i}=\overset{n}{\underset{i=1}{\sum }}w_{k}\times T \end{array}$$

After calculating the brand conversion intention, the uncertainty of conversion intention is caused by the influence of sales volume. Based on the results of cluster analysis, we set the threshold values of monthly sales and switching intention, and divided the brand switching types into four categories.

Specifically, x and y are input to two feature pyramid networks, which use convolutional neural networks to extract multi-scale deep features of different classes of images, and then the extracted features are further sub-represented in the shared domain S as x, y ϵ R (H, W are feature space dimensions, C is the channel direction dimension) by the dense correspondence module. Then the domain alignment can be expressed by the following equation:


(9) $$\begin{array}{l} x=ℑ\|l\left(t\right)-q\| \end{array}$$

(10) $$\begin{array}{l} y=ℑ\|x\left(t\right)-q\| \end{array}$$

Specifically, the correlation matrix M, where each element is correlated in pairs of features, is calculated. The correlation matrix M is calculated as shown in the following equation:


(11) $$\begin{array}{l} M\left(x,y\right)=\frac{{\left[x\left(u\right)-y\left(u\right)\right]}^{T}\left[x\left(u\right)-y\left(u\right)\right]}{{\left[\left\|x\left(u\right)-y\left(u\right)\right\|\right]}^{T}\|x\left(u\right)-y\left(u\right)\|} \end{array}$$

An effective way to do this is to train the cross-domain correspondence network together with the image enhancement network.

## Example Calculation Results and Analysis

### Data Collection and Calculation

Taking the headphone brand as an example, we extracted a total of 7,857 reviews of 10 brands by using web crawler software to crawl the relevant review index information, and 6,482 reviews were extracted for calculation after deleting duplicate and invalid reviews. The ECM-ISC model was used to calculate the feature weights of the four factors using the formula, and the factor weights of the four indicators were: W = (0.295, 0.204, 0.151, 0.350). According to the formula, the expectation confirmation degree, perceived value, contributing factors and satisfaction of each brand were calculated. According to the fuzzy calculation and inference rules of brand switching intention, the fuzzy calculation results of the four antecedent factor indicators and brand switching intention in the actual reviews were calculated as shown in Table 2.

**Table 2:** Mobile phone brands and their calculation results.

**Serial number**	**Brands**	**Model**	**Release Time**	**Price (yuan)**	**Bluetooth version**
1	Apple	AirPods National	42,621	1,276	Undisclosed
2	Anker	Soundcore LIBERTY AIR	43,397	699	5
3	BOSE	SOUNDSPORT FREE	43,070	1,998	
4	BandO	Beoplay E8	42,948	1,972	4.2
5	BRAGI	The Headphone TWS	42,614	1,129	4.2
6	Crazybaby	Air by Crazybaby	42,917	688	4.2
7	Dacom	K6H	43,221	199	4.1
8	EDIFIER	TWS3	43,252	398	4
9	EDIFIER	TWS2	43,374	299	5
10	Crazy Rice	FUNCL AI	43,405	299	5

### Validity Analysis

**Figure 2:**
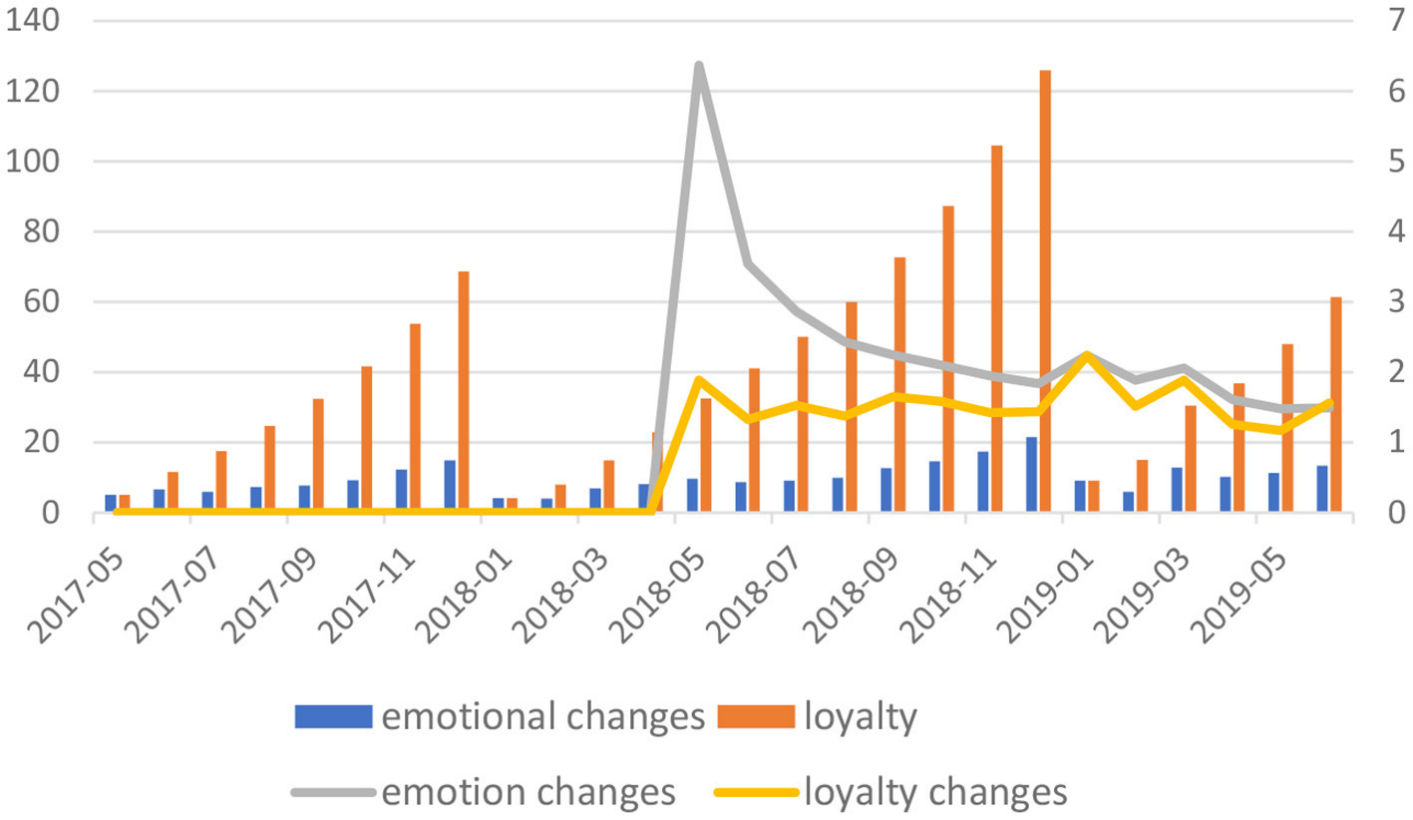
Loyalty type identification.

The online reviews of the four brands were extracted and the perceived functional value, perceived symbolic value and emotional value were calculated according to the fuzzy calculation steps, and the results of the calculations obtained by the fuzzy inference system according to the consumer brand loyalty inference rules are shown in Figure 2. From the data analysis we can see that, the perceived functional value of the other three brands has a positive correlation change with price. The higher the price of the product, the higher the perceived usefulness of the product, i.e., the performance of the cosmetic. This confirms the common saying that “you get what you pay for”. However, for products that do not vary much in value at the same price, consumers perceive a different change in functional value. This also shows that consumers' perceived value of the brand is not a single trade-off of interest. According to the rules of emotion lexicon construction, the dynamic emotion words, value evaluation words and emotion modification words in the reviews conforming to the rules of fuzzy corpus were extracted by manual and Matlab2014a data pre-processing, and the extracted words were semantically analyzed, and then the emotion words of 8 cell phone brands were extracted for attribute classification by manual analysis, screening and noise removal.

Overall, the symbolic value of all four cosmetic products at different price points is positively correlated with the price of the cosmetic product. That is, price influences the symbolic value of the product as perceived by consumers. The level of consumption reflects the status of the consumer. High-end cosmetic brands have a high symbolic value due to their brand identity and fashionability, which is far beyond the reach of mid- and low-end brands. This reflects the underlying need for social identity, self-expression, socialization and self-esteem. Brand reputation, brand image and brand fashionability are all important to consumers seeking symbolic value.

Based on the research of relevant theoretical foundations at home and abroad, this paper clarifies the main influencing factors of consumer brand loyalty and constructs a computable model. And identifies group consumer loyalty of brands based on online reviews with text mining technology and semantic fuzzification calculation. Using fuzzy algorithm implementation, inference rules are established and fuzzy inference is performed with examples of Taobao species in different price ranges. The quantitative calculation of brand group consumer loyalty is realized. The experimental results are more satisfactory and basically verify the accuracy of the model and calculation method. The method is provided for the computability of brand loyalty. For the analysis and research of the example results, this facilitates the scientific and refinement of marketing management, and then improves the marketing strategy and other aspects with important practical significance. The virtual brand community is characterized by information exchange, where customers obtain information about brands or products from the virtual brand community by browsing or asking, and enterprises obtain demand information from customers to ensure the consistency of customer needs and enterprise value proposition. Co-production, where customers participate in various design and innovation idea solicitation activities organized by enterprises in the virtual brand community. Interpersonal interaction, where customers interact with other members of the virtual interpersonal interaction, where customers interact with other members of the community or company staff in the virtual brand community.

Based on the connotation theory of brand loyalty and the study of brand loyalty classification, the classification of consumer loyalty types based on online reviews is proposed. By extracting the sentiment values from online reviews, the consumer sentiment values are calculated using fuzzy through the processes of semantic fuzzification and semantic integrated fuzzy calculation. Comprehensive brand loyalty as well as sentiment value are analyzed, and it is concluded that brand loyalty of consumers based on online reviews is divided into: disloyalty, false loyalty, potential loyalty and sustained loyalty. In addition, a computable model of brand switching intention is constructed in this paper. This paper provides a method for calculating group consumer loyalty and brand switching intention based on mining text semantics of online reviews, calculating semantic emotions, and using fuzzy calculation and inference methods, and validates it with a review example study. Based on the research results, companies can better understand consumers' brand loyalty and brand switching motives, and then develop effective marketing strategies in a targeted manner. However, there are also some limitations in the study, such as the lack of detail in the establishment of inference rules and the lack of perfect construction of the corpus, which can be further improved in the future analysis.

### Data Clustering and Analysis

**Figure 3:**
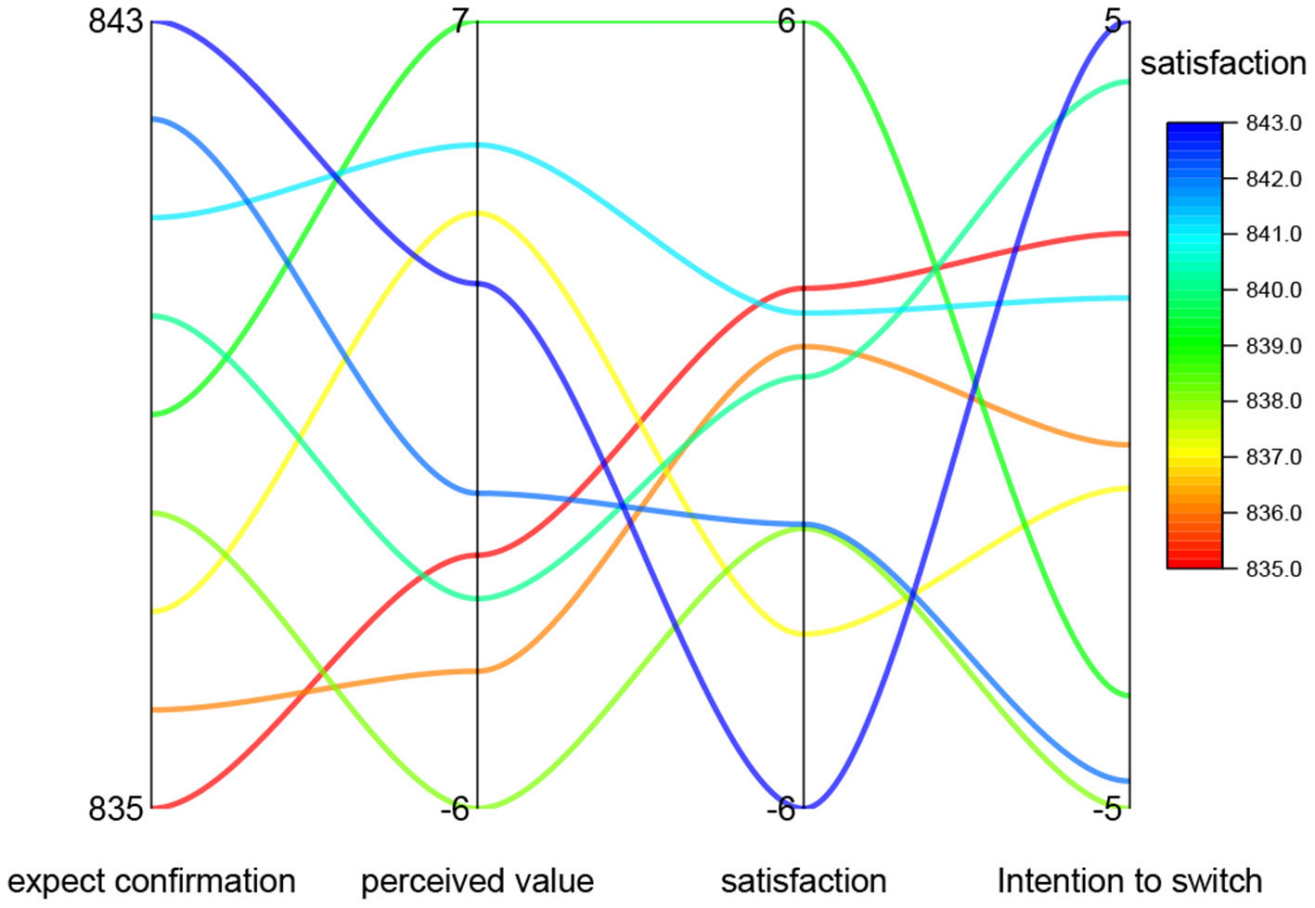
Brand switching intentions relationship chart.

The two-dimensional relationship between expectation confirmation, perceived factors, contributing factors, satisfaction and brand switching intention for each cell phone brand through fuzzy data mining of cell phone brand online reviews is shown in Figure 3. In this study, 25.9% of the respondents were female and 74.1% were male, and the proportion of males was much larger than that of females, probably because the sample mainly came from virtual brand communities of electronic products, and most of the consumers in these communities were mainly male. The age of the respondents was mainly distributed between 18 and 30 years old, and overall the respondents were relatively young, probably because the subjects of this study were virtual brand communities. In terms of education structure, most of the respondents' education level is college or above, which accounts for 56.6% of the total sample. It indicates that the respondents have higher education level, which is also consistent with the target of this study.

**Figure 4:**
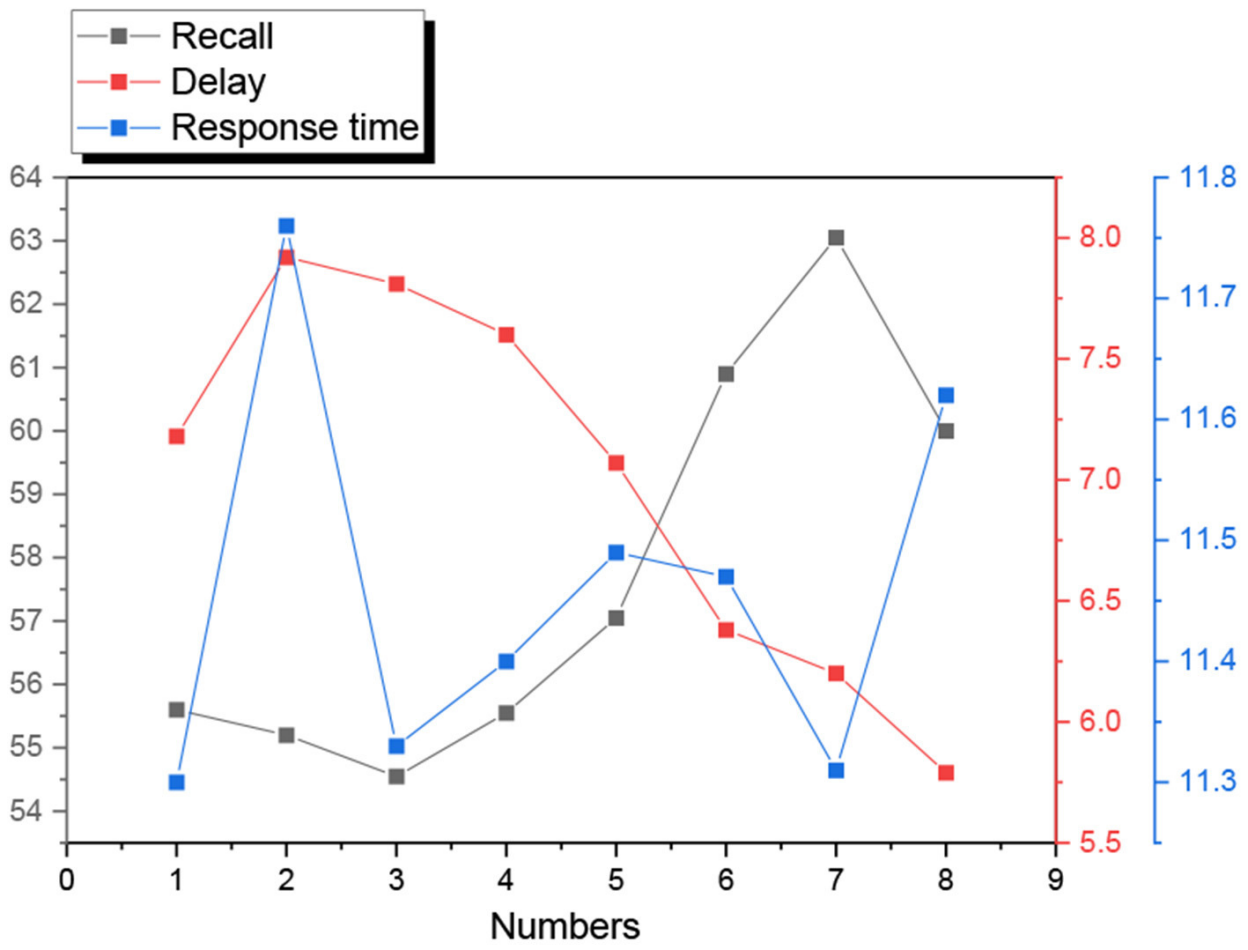
Clustering results of brand switching intention.

The two-dimensional relationship diagram of IF-THEN model data inference shows that there is a great similarity in the trends of the same clusters of cell phone brand expectation confirmation, perception factors, contributing factors, and satisfaction, while there are great differences in cell phone brand switching intention, so the model data can be clustered. From a statistical point of view, cluster analysis is a method to simplify data categorization through data modeling. The clustering results for cell phone brand switching intention are shown in Figure 4. According to the model 1 of the direct effect of customer experience value on customer commitment, it is verified that customer experience value has a direct and significant positive effect on customer commitment, which satisfies the first condition of the mediating effect. The second condition of the mediating effect is satisfied. according to the partial mediating effect model 3 of customer satisfaction, it is verified that customer experience value has a significant positive influence on customer satisfaction and customer satisfaction has a significant positive influence on customer commitment, but the path coefficient of the influence of customer experience value on customer commitment is 0.373, which is smaller than the path coefficient of the direct influence of customer experience value on customer commitment, which is 0.841, indicating that the relationship between customer satisfaction in customer. This indicates that customer satisfaction plays a part of mediating effect in the relationship between customer experience value and customer commitment. Therefore, hypothesis H6 is accepted.

Consumers' perceived value has a positive influence on brand loyalty, and a library of brand loyalty inference rules is constructed, as shown in Table 3. Before establishing the rules, in order to facilitate the establishment of the inference rules and the analysis of the results, the range of perceived functional value and perceived symbolic value of consumers is divided into the previous paper. In order to achieve more clarity in the results, both perceived functional value and perceived symbolic value were extended in scope and increased in level by a factor of one, with the scope extended from to. The level of each was also increased accordingly. The same proportion is enlarged to one level in the positive direction, one level in the non-polar direction, and one level in the negative direction. And in the actual calculation, we calculate the actual range of perceived functional value, perceived symbolic value, and emotional value as that. Because if the perceived functional value, perceived symbolic value and emotional value have negative values, it means that most consumers think that the brand is very bad and they are against the brand and refuse to buy it, so obviously such a brand cannot survive in the fierce market competition.

**Table 3:** Comparison of different intelligent algorithms.

**Algorithms**	**Algorithm accuracy (%)**	**Time (s)**
Genetic algorithm	82	12.91
Ant colony algorithm	84	10.07
Neural network algorithm	76	10.69
Fuzzy emotion algorithm	68	12.13

The best strategy for long-term corporate brand positioning is “imitation symbiosis”, that is, providing similar benefit points. The benefit point also reflects the degree of similarity of benefit points of different corporate brands, for corporate brand 2X: the larger the nm, i.e., the more similar the benefit points are, the greater the benefit, and the maximum benefit when the benefit points are identical (m = 1 and *n* = 1) and equal to the benefit of corporate brand 1X. The “imitation symbiosis” reflects the importance of learning and imitation for long-term corporate brand positioning. Point-of-interest substitution leads to a reduction in the market size of the category. When the category is monopoly-free, the points of interest are to some extent substitutable, which leads to the effective supply being smaller than the actual supply, and the sum of the returns of the two brands being smaller than the market size of the entire category. In the long run, corporate brands can maximize their returns simultaneously, except when there is no monopoly (m = 1 and *n* = 1). When the positioning point is the same (the supply decision is the same y), each corporate brand can obtain the maximum revenue at the same time, and as long as the corporate brand positioning strategy is appropriate, it can achieve an infinite number of equilibrium states, in which each corporate brand supply decision is the same and all achieve the maximum revenue. When a company's brand positioning changes, another company's brand can achieve a new equilibrium simply by making corresponding adjustments.

### Correlation Analysis

**Figure 5:**
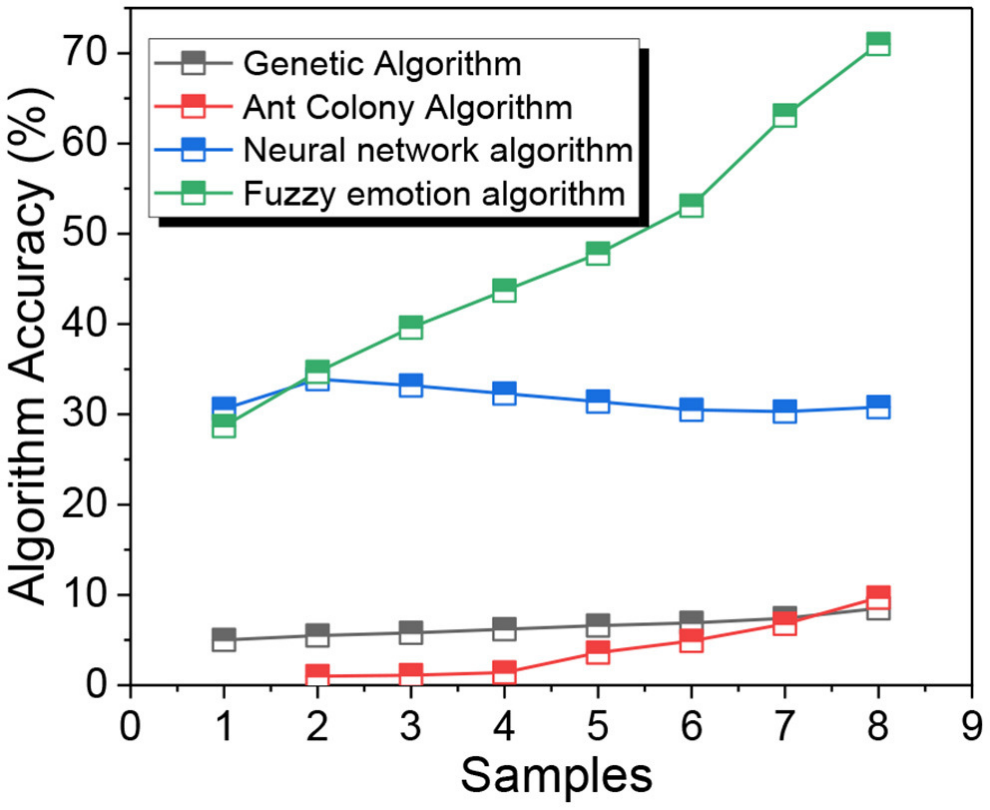
Comparison of accuracy among different algorithms.

The results of this paper are shown in Figure 5. Based on the clustering results and Gnaesh's brand segmentation theory, the critical values of monthly sales and conversion intention are set to 2000 and 3.0 respectively. When the positioning of a corporate brand changes, another corporate brand can achieve a new equilibrium state with appropriate positioning point adjustment, and each corporate brand can obtain the maximum benefit in the equilibrium state. Positioning duration affects corporate brand revenue. In the short term, corporate brands cannot maximize their revenue at the same time, and there is no situation in which both corporate brands can maximize their revenue at the same time. In the long term, corporate brands can maximize revenue at the same time, and each corporate brand can maximize revenue at the same time when the positioning points are consistent. Positioning duration affects corporate brand behavior. In the short term, corporate brands can only passively accept the existing situation in time to adjust, and the best strategy for corporate brand positioning is to enter the opposite market. In the long term, corporate brands can take self-interested actions and constantly adapt to adjustments, when the best strategy for corporate brand positioning is “imitation symbiosis”, that is, providing similar benefits.

The initial positioning point affects the category characteristics. When different brands can provide the same or similar points of interest, the category market is non-monopolistic, and the brands can replace each other. When a brand can provide unique points of interest, the category market is monopolistic, and the brand is irreplaceable. Dynamic positioning point affects corporate brand revenue. Positioning points are the points of interest chosen or emphasized by a corporate brand, and they are reflected in the model as market entry situations, with different market entry decisions corresponding to different benefits. In addition, there are numerous equilibrium states for long-term corporate brand positioning.

The content of corporate brand communication is the information to be expressed by corporate brand communication. The content of corporate brand communication is rich and diverse, including both corporate news and product claims, including both market PR and marketing advertising, and can be the enterprise itself and social welfare. In the final analysis, the content of corporate brand communication can be divided into two types: corporate brand visual and corporate brand culture, of which corporate brand visual is the visible part and corporate brand culture is the invisible part. The visible corporate brand communication content is the corporate brand vision. Visual signs and symbols are important sources for people to obtain corporate brand information. Corporate brand vision is an important reflection of corporate brand culture, a prerequisite for corporate brand to play its information function, a carrier of corporate brand information, a visual expression of corporate brand communication, and a visual presentation of corporate culture. The invisible corporate brand communication content is the corporate brand culture. Culture is the driving force of the internal development of the enterprise. Cultural factors play a central role in the formation of the enterprise brand, a pioneering role in the innovation of the enterprise brand, and a bridging role in the communication of the enterprise brand. A strong corporate brand must have a strong corporate brand culture. Corporate brand visuals can be changed flexibly, while corporate brand culture is relatively stable. Therefore, corporate brand culture communication is to express a more unified connotation (corporate brand culture) through diverse contents (corporate brand visuals).

## Conclusion

With increasingly competitive markets, consumer brand loyalty is the most competitive resource for companies, and brand loyalty is the most valuable and abundant asset. Consumers' loyalty to brands is a business goal that companies must pursue. Exploring the reasons for the formation of brand loyalty and distinguishing the types of consumer loyalty are fundamental for companies to ensure sustainable development. This paper combines brand loyalty theory and perceived value theory to classify brand loyalty into two dimensions: perceived functional value and perceived symbolic value. The functional evaluation words and symbolic evaluation words in online reviews are extracted, the semantic analysis and sentiment calculation of the evaluation words are carried out, a fuzzy ontology library is constructed, the principle of fuzzy inference is used, and the perceived functional and perceived symbolic values are used as inference antecedents to fuzzy infer the magnitude of brand loyalty, which provides a new method for the quantitative calculation of brand loyalty of group consumers. Semantic mining and data analysis of online product reviews is an important issue in current e-commerce intelligence analysis in the context of big data. From the filtering results, it can be seen that the recall and accuracy of the tested model reach a satisfactory level, especially for the persistent conversion and unsatisfactory conversion measures. This shows that the constructed model has a good effect on the determination of brand switching intention of online review users. Based on the fuzzy sentiment of online reviews, this paper improves the continuous use model ECM-ISC and formulates the inference rules of fuzzy affiliation function, and verifies the brand conversion intention and brand conversion type of cell phones by example calculation, which has good accuracy and generality and has important practical significance for brand marketing and early warning management. At the same time, there are some limitations in this paper, such as the complexity of the fuzzy corpus rules, which makes the comment statistics rely on manual identification and classification, and there is also room for improvement in the sentiment polarity judgment and analysis methods. In the future, we will further enrich the corpus sentiment lexicon to improve the accuracy of sentiment word analysis and the scientificity of sentiment polarity judgment.

## Data Availability Statement

The original contributions presented in the study are included in the article/supplementary material, further inquiries can be directed to the corresponding author.

## Author Contributions

The author confirms being the sole contributor of this work and has approved it for publication.

## Funding

This work was supported by School of Media and Communication, Shanghai Jiao Tong University.

## Conflict of Interest

The author declares that the research was conducted in the absence of any commercial or financial relationships that could be construed as a potential conflict of interest.

## Publisher's Note

All claims expressed in this article are solely those of the authors and do not necessarily represent those of their affiliated organizations, or those of the publisher, the editors and the reviewers. Any product that may be evaluated in this article, or claim that may be made by its manufacturer, is not guaranteed or endorsed by the publisher.
